# Anemia among Chinese patients with chronic kidney disease and its association with quality of life - results from the Chinese cohort study of chronic kidney disease (C-STRIDE)

**DOI:** 10.1186/s12882-021-02247-8

**Published:** 2021-02-22

**Authors:** Yan Shen, Jinwei Wang, Jing Yuan, Li Yang, Fangfang Yu, Xiaolei Wang, Ming-Hui Zhao, Luxia Zhang, Yan Zha, the Chinese Cohort Study of Chronic Kidney Disease (C-STRIDE), Ming-Hui Zhao, Luxia Zhang, Xiaoqin Wang, Jun Yuan, Qiaoling Zhou, Qiongjing Yuan, Menghua Chen, Xiaoling Zhou, Shuxia Fu, Shaomei Li, Yan Zha, Zhangsuo Liu, Jun Jun Zhang, Li Wang, Lei Pu, Jian Liu, Suhua Li, Zuying Xiong, Wei Liang, Jinghong Zhao, Jiao Mu, Xiyan Lian, Yunjuan Liao, Hua Gan, Liping Liao, Rong Wang, Zhimei Lv, Yunhua Liao, Ling Pan, Xiaoping Yang, Zhifeng Lin, Zongwu Tong, Yun Zhu, Qiang He, Fuquan Wu, Rong Li, Kai Rong, Caili Wang, Yanhui Zhang, Yue Wang, Wen Tang, Hua Wu, Ban Zhao, Rongshan Li, Lihua Wang, Detian Li, Feng Du, Yonggui Wu, Wei Zhang, Shan Lin, Pengcheng Xu, Hongli Lin, Zhao Hu, Fei Pei, Haisong Zhang, Yan Gao, Luying Sun, Xia Li, Wenke Wang, Fengling Lv, Deguang Wang, Xuerong Wang, Dongmei Xu, Lijun Tang, Yingchun Ma, Tingting Wang, Ping Fu, Tingli Wang, Changying Xing, Chengning Zhang, Xudong Xu, Haidong He, Xiaohui Liao, Shuqin Xie, Guicai Hu, Lan Huang

**Affiliations:** 1 Department of Nephrology, Guizhou Provincial People’s Hospital, Guizhou University School of medicine, Gui Yang, China; 2 Renal Division, Department of Medicine, Peking University First Hospital, Peking University Institute of Nephrology; Key Laboratory of Renal Disease, Ministry of Health of China; Key Laboratory of Chronic Kidney Disease Prevention and Treatment (Peking University), Ministry of Education, No. 8 Xishiku Street, Xicheng District, Beijing, China; 3 Department of Statistics, University of Michigan, 1085 South University, Ann Arbor, MI USA; 4 Peking-Tsinghua Center for Life Sciences, Beijing, China; 5 National Institute of Health Data Science at Peking University, No. 38 Xueyuan Street, Haidian District, Beijing, China; 6 Center for Data Science in Health and Medicine, Peking University Health Science Center, No. 38 Xueyuan Street, Haidian District, Beijing, China

**Keywords:** Anemia, Chinese patients, Chronic kidney disease, Quality of life - results, C-STRIDE

## Abstract

**Background:**

Anemia is one of the common complications in patients with chronic kidney disease (CKD). However, there is no systematic investigation on the prevalence of anemia in CKD patients and its relationship with the quality of life in China.

**Methods:**

The data for this study comes from baseline data from the Chinese Chronic Kidney Disease Cohort Study (C-STRIDE), which recruited predialysis CKD patients in China. The kidney disease quality of life summary (KDQOL-TM) was used to assess health-related quality of life (HRQoL). Use linear regression model to estimate the relationship between hemoglobin level and quality of life.

**Results:**

A total of 2921 patients were included in this study. The adjusted prevalence of hemoglobin (Hb) less than 100 g/L was 10.3% (95% confidence interval [CI]: 9.9,11.4%), and showed an increased trend through reduced eGFR levels from 4.0% (95%CI:2.3,5.9%) in the 45-60 ml/min/1.73m^2^ group to 23.4% (95%CI:20.5,26.2%) in the 15–29 ml/min/1.73m^2^ group. The prevalence of anti-anemia treatment was 34.0% (95%CI: 28.7,39.3%) and it is shown by reducing eGFR levels from 15.8% (95%CI:0,36.7%) in the 45-60 ml/min/1.73m^2^ group to 38.2% (95%CI: 30.7,45.2%) in the 15–29 ml/min/1.73m^2^ group. All five dimensions of the KDQOL scores in patients with CKD decreased as hemoglobin declined. After multivariable adjustments,the degrees of decrease became somewhat blunted. For example, compared with hemoglobin of ≥130 g/L, regression coefficients in the hemoglobin of < 100 g/L were − 0.047(95%CI: − 0.049,-0.045) for Symptoms and Problems(S), − 0.047(95%CI: − 0.049,-0.044) for Effects of the Kidney Disease(E), − 0.207(95%CI: − 0.212,-0.203) for Burden of the Kidney Disease(B), − 0.112(95%CI: − 0.115,-0.109) for SF-12 Physical Functioning (PCS), − 0.295(95%CI: − 0.299, -0.292) for SF-12 Mental Functioning (MCS), respectively.

**Conclusions:**

In our cross-sectional analysis of patients with CKD in China, prevalence of both anemia and anti-anemia treatment increased with decreased eGFR. In addition, anemia was associated with reduced HRQoL.

## Background

Chronic kidney disease (CKD) is highly prevalent in China, which has been recent estimates indicating that up to 10.8% of people aged 18 years or older have the disease [1]. Anemia commonly occurs in people with chronic kidney disease (CKD) [2–4]^.^ In the United States, the prevalence of anemia in CKD patients was 15.4%, which is increased with stage of CKD, from 8.4% at stage 1 to 53.4% at stage 5 [5]. In the research work conducted in Shanghai, China, the total prevalence of anemia was 51.5% in CKD patients with 22.4% in stage 1, 30.0% in stage 2, 51.1% in stage 3, 79.2% in stage 4, and 90.2% in stage 5, respectively.,which also reported that 44.9% patients has being treated for anemia [6]. With the progress of CKD stages, the treatment rate of anemia increased,19.4% in stage 1, 11.4% in stage 2, 26.9% in stage 3, 46.3% in stage 4, and 73.0% in stage 5, respectively. However, in total chinese population the current prevalence of anemia is still unknown.

According to recent clinical guidelines, health-related quality of life (HRQoL) is an important key indicator for CKD anemia management [7, 8]. HRQoL provides a comprehensive appraisal of disease burden, incorporating assessment of symptoms, functional capacity, and well-being related to an effective treatment. A recent systematic review [9] showed that treatment of anemia comparing with untreated anemia among patients with CKD was associated improvement of HROoL. However, the aimed of higher hemoglobin level may not be necessary, which has a connection with a certain outcome of HROoL improvement. In addition, Wyatt et al [10] reported some individual patients may benefit in their subjective overall well-being from slightly higher hemoglobin levels in the range of 115–130 g/l, but the parameters were difficult to quantify.

This study is a multi-center prospective cohort study and the first national CKD cohort study in China. We aim to provide the prevalence of different hemoglobin levels and the treatment of anemia in Chinese CKD patients, and to explore the relationship between different hemoglobin levels and quality of life .

## Methods

### Study design

The Chinese Cohort Study of Chronic Kidney Disease (C-STRIDE) is an ongoing multicenter prospective cohort and the first national CKD cohort in China, which contains 39 clinical centers located on 28 cities in 22 provinces in China. Screening visit was conducted by nephrologists at each clinical center. The design and methods of the Chinese Cohort Study of Chronic Kidney Disease (C-STRIDE) study were published in detail already [11]. This study is a cross-sectional analysis based on the baseline data of C-STRIDE.

The C-STRIDE study was conducted in accordance with the Declaration of Helsinki. The study has been approved by the ethics committee of Peking University First Hospital (Approval Number: 2011[363]). All participants provided informed consent.

### Measurements

Ascertainment of Level of Kidney Function- Kidney function was quantified using eGFR derived from the four-variable Modification of Diet in Renal Disease (MDRD) study equation [12].

Hemoglobin- Serum hemoglobin (HGB) was measured using a coulter LH 750 Hematology Analyzer (Beckman Coulter, Brea, CA,USA). The patients were divided into four groups according to the hemoglobin level at enrollment: hemoglobin level < 100 g/L, hemoglobin level 100 –115 g/L, hemoglobin level 116 –129 g/L, hemoglobin level ≥ 130 g/L.

HRQoL was assessed using KDQOL-TM at the same time [13], The Mandarin Chinese version of kidney disease quality of life (KDQoL)-36 instrument, a globally accepted tool for evaluating HRQoL in patients with CKD [8]. The disease-related section consisted of 24 items which made up three scales: Symptoms and Problems (12 items), Burden of Kidney Disease (4 items), and Effects of Kidney Disease (8 items). The generic core was the 12-item Short-Form Health Survey (SF-12). The results of the SF-12 instrument were summarized into the Physical Component Summary (PCS) score and the Mental Component Summary (MCS) score. The raw scores were transformed linearly into a range of 0–100 with higher scores representing greater HRQoL [10] .

Other laboratory measurements and patient characteristics were in contained as well, including demographics (age, gender, education, income), lifestyle (smoking), medical history (diabetes, anemia treatment: ESAs, iron [include oral and IV], or ESA combined with iron. Hypertension treatment physical examination findings (height, weight, body mass index, systolic blood pressure and diastolic blood pressure), urinary albumin-creatinine ratio (UACR) and left ventricular hypertrophy (LVH).

A two-dimensional guided M-mode echocardiographic study was performed at each nephrology center. Measurements included the diastolic thickness of the interventricular septum (IVST), left ventricular posterior wall (PWT), and the internal diameter of the left ventricle at the end of diastole (LVDd). Left ventricular mass (LVM) was calculated by using the formula: LVM = 0.8 (1.04 [LVDd+IVST+PWT] 3 – [LVDd] 3) + 0.6 [14]; left ventricle mass index (LVMI) was measured by dividing left ventricle muscle mass to body surface area (BSA). BSA = 0.0061 × height (cm) + 0.0128 × weight(kg) − 0.1529. LVMI > 125 g/m^2^ in males and > 120 g/m^2^ in females were considered as LVH.

### Statistical analysis

Continuous data are presented as means with standard deviations (SDs) except for UACR, which is presented as median (inter-quartile range, IQR) because of skewed distribution of data. Categorical variables are presented as proportions. Hemoglobin was divided into four groups with the levels of < 100 g/L,100- 115 g/L,116-129 g/L, and ≥ 130 g/L, respectively. Relevant characteristics were described and compared according to different hemoglobin levels. One-way ANOVA or Kruskal-Wallis test was used for comparison of continuous variables and the chi-square test for binary variables between hemoglobin levels. The prevalence of anemia based on different Hb levels and the use of anemia treatments of anemia are reported among total population and different eGFR levels.

Hemoglobin was analyzed as a categorical variable with 4 categories in the regression models and the hemoglobin of ≥130 g/L was used as a reference when comparing the difference in KDQOL scores among different hemoglobin groups. Generalized linear models were used to test the effects of the independent variables on the KDQOL scores. Each adjusted mean difference from the reference group was computed based on the estimated marginal means of the KDQOL scores. Starting with an unadjusted model, we sequentially introduced blocks of variables to evaluate their effects on the association between hemoglobin and HRQoL. Model 1 was unadjusted, model 2 was adjusted for social-demographics (age, sex, education, income), model 3 was additionally adjusted for cardiovascular risk factors (smoking, BMI, hypertension, and diabetes), and model 4 was further adjusted for comorbid conditions (LVH). The marginal means were estimated as the mean value averaged of all cells generated by the rest of the categorical variables, with the value of each covariate set to its overall mean estimate. Statistical analyses were performed with SAS (version 9.4; SAS institute, Cary, NC). *P* value of < 0.05 in two-sided test was considered as statistical significance.

## Results

Altogether, 3499 Chinese urban patients with non-dialysis CKD stage 1–4 were enrolled in this study. We excluded 578 participants due to missing key demographic variables (serum creatinine or hemoglobin level), resulting in 2921 patients included in the analysis. The mean age of patients was 48.5 ± 13.6 years, and 1710 (58.5%) were male, of which 2016 (69.0%) patients had an eGFR less than 60 mL/min per 1.73 m^2^. The mean hemoglobin level of the patients was 127.8 ± 21.9 g/L. When stratified by four stages of hemoglobin levels, there were significant differences in age, gender,diabetes, education level,income, UACR levels,blood pressure, BMI,albumin, ACR,eGFR; The adjusted prevalence of hemoglobin (Hb) less than 100 g/L was 10.3% (95% confidence interval [CI]: 9.9,11.4%) and showed an increased trend through reduced eGFR levels from 4.0% (95%CI:2.3,5.9%) in the 45-60 ml/min/1.73m^2^ group to 23.4% (95%CI:20.5,26.2%) in the 15–29 ml/min/1.73m^2^ group.

We presented the prevalence of anemia treatment based on hemoglobin level of patients and CKD stages with reduced kidney function. Among those with hemoglobin< 100 g/L, the prevalence of anemia treatment was 34.0% (95%CI: 28.7–39.3%). The prevalence of treatment with ESA only and iron only was similar, which was 24.0% (95%CI: 19.3–29.0%) and 24.7% (95%CI: 19.7–29.3%), respectively, and the prevalence of treatment with combined usage was 14.7%. An increased trend for anemia treatment was observed with reduced levels of eGFR. For example, the patients of CKD stage 3a, 3b and 4 with hemoglobin< 100 g/L, 15.8, 32.8 and 38.2% were receiving anemia treatment,, respectively. Similar patterns can be found in the group of the treatment with ESA and iron. To be noted, only 5.3% of ESA-treated patients in CKD Stage 3a were observed to be with hemoglobin< 100 g/L and 5.2% patients with hemoglobin 100-115 g/L treated with ESA in CKD stage 3a, compared to the prevalence of patients treated with iron was 15.8 and 13.8%, respectively (Table 3).

Among 2921 patients, 2333 (79.9%) received baseline echocardiography, of which 227 (7.8%) were eligible for LVM assessment. As hemoglobin levels decrease, the prevalence of left ventricular hypertrophy increases. The ratio of more than 100 g/L was 18.3% (95% CI: 9.9–11.4%), and it showed a significant downward trend with the increase of hemoglobin level.

As shown in Table 1, the HRQoL scores in all dimensions impaired progressively and significantly (*p* < 0 .001) across hemoglobin level. The lowest scores were found in hemoglobin< 100 g/L. Scores across all the five dimensions of the KDQOL included Symptoms and Problems (S), Effects of the Kidney Disease (E), Burden of the Kidney Disease (B), SF-12 Physical Functioning (PCS), SF-12 Mental Functioning (MCS) were 81.2 ± 23.5, 62.7 ± 26.6, 72.9 ± 19.7, 86.8 ± 13.0, 74.1 ± 27.1, respectively. Scores of KDQOL were negatively associated with the decreased levels of hemoglobin. The strength of association increased through the decreased levels of hemoglobin.

**Table 1:** Baseline Characteristics of participants according to hemoglobin levels

Characteristic	Total(*N* = 2921)	< 100(*n* = 300)	100–115(*n* = 525)	116–129(*n* = 716)	≥130(*n* = 1380)	Missing value	*p*-value
Age (years)	48.5 ± 13.6	52.8 ± 13.0	51.2 ± 13.1	50.8 ± 13.6	45.5 ± 13.4	0	< 0.001
Men (%)	1710(58.5)	129(7.5)	199 (11.6)	312 (18.2)	1070 (62.7)	0	< 0.001
eGFR	52.3 ± 33.4	29.9 ± 22.5	38.2 ± 27.8	49.9 ± 31.4	63.7 ± 33.6	0	< 0.001
Prevalence (%)(95%CI)		10.3 (9.2,11.4)	18.0 (16.6,19.4)	24.5 (23.0,26.1)	47.2 (45.4,49.1)		
≥ 60(CKD1–2)		2.7 (1.5,3.6)	8.0 (6.2,9.7)	21.1 (18.3,23.8)	68.3 (65.4,71.4)		
45–59(CKD3a)		4.0 (2.3,5.9)	12.1 (9.4,15.3)	24.5 (20.7,28.2)	59.4 (54.8,63.6)		
30–44(CKD3b)		8.4 (6.3,10.5)	19.5 (16.6,22.5)	27.8 (24.4,31.4)	44.3 (40.6,48.3)		
15–29 (CKD4)		23.4 (20.5,26.2)	30.7 (27.7,34.0)	25.5 (22.6,28.7)	20.4 (17.4,23.2)		
Hemoglobin(g/L)	127.8 ± 21.9	89.3 ± 8.0	108.4 ± 4.5	122.7 ± 4.0	146.3 ± 12.7	0	0.001
Diabetes (%)	393 (13.5)	100 (25.4)	97 (24.7)	101 (25.6)	95 (24.2)	48	0.056
≥High school education (%)	1625 (55.6)	125 (7.7)	240 (14.8)	381 (23.4)	879 (54.1)	28	< 0.001
Income, yuan(%)						123	< 0.001
< 30,000	997 (34.1)	136 (13.6)	176 (17.7)	249 (25.0)	436 (43.7)		
30,000–50,000	726 (24.9)	75 (25.0)	157 (29.9)	171 (23.9)	323 (23.4)		
50,000–100,000	1075 (41.0)	89 (29.7)	192 (36.6)	296 (41.3)	621 (45.0)		
Current smoker (%)	1079 (36.9)	85 (28.3)	147 (28.0)	209 (29.2)	638 (46.2)	62	0.72
Use anemia drug(%)	323 (11.1)	102 (34.0)	118 (22.5)	80 (11.2)	23 (1.7)	0	< 0.001
EPO	206 (7.1)	72 (24.0)	75 (14.3)	44 (6.1)	15 (1.1)		
Iron	234 (8.0)	74 (24.7)	87 (16.6)	57 (8.0)	16 (1.2)		
EPO + Iron	117 (4.0)	44 (14.7)	44 (8.4)	21 (2.9)	8 (0.6)		
SBP (mm Hg)	130.0 ± 18.8	135.8 ± 19.7	131.9 ± 17.4	128.3 ± 17.3	126.9 ± 16.2	178	< 0.001
DBP (mm Hg)	81.1 ± 11.7	80.3 ± 10.5	80.0 ± 10.5	79.7 ± 10.2	81.4 ± 10.6	178	0.001
BMI (kg/m^2^)	24.6 ± 3.8	24.2 ± 4.1	23.4 ± 3.6	24.4 ± 3.6	25.2 ± 3.9	80	< 0.001
LHV(%)	227 (7.8)	55 (18.3)	55 (10.5)	54 (7.5)	63 (4.6)	427	< 0.001
ACR (mg/g creatinine)	418.3 (113.8985.3)	718.5 (219.91676.6)	554.3 (181.0,1226.6)	410.0 (98.0,952.5)	349.8 (105.1814.2)	407	< 0.001
Use antihypertensive drugs(%)	463 (15.9)	147 (49.0)	107 (20.4)	104 (14.5)	105 (7.6)	0	< 0.001
ACEI/ARB	324 (11.1)	93 (31.0)	69 (13.1)	81 (11.3)	81 (5.9)		
CCB	361 (12.4)	120 (40.0)	80 (15.2)	84 (11.7)	77 (5.6)		
ALB(g/L)	38.9 ± 7.3	34.9 ± 7.5	38.0 ± 7.1	38.6 ± 7.0	40.2 ± 7.1	130	< 0.001
Hs-CRP (mg/L))	3.7 ± 12.1	5.3 ± 12.0	5.3 ± 6.8	5.7 ± 9.9	5.7 ± 5.8	288	0.038
TC (mmol/L)	5.5 ± 7.0	4.6 ± 2.0	4.4 ± 15.9	3.3 ± 7.8	3.3 ± 12.2	196	0.075
Symptoms and Problems (S)	81.2 ± 23.5	72.8 ± 27.5	77.5 ± 25.3	81.9 ± 22.5	84.0 ± 21.8	395	< 0.001
Effects of the Kidney Disease (E)	62.7 ± 26.6	55.5 ± 26.7	58.7 ± 27.4	63.4 ± 27.1	65.4 ± 25.5	395	< 0.001
Burden of the Kidney Disease (B)	72.9 ± 19.7	66.9 ± 22.4	70.1 ± 20.1	72.6 ± 19.7	75.4 ± 18.2	395	< 0.001
SF-12 Physical Functioning (PCS)	86.8 ± 13.0	81.0 ± 16.5	84.5 ± 12.5	86.7 ± 13.1	89.0 ± 11.2	395	< 0.001
SF-12 Mental Functioning (MCS)	74.1 ± 27.1	60.6 ± 30.8	69.7 ± 27.0	73.7 ± 26.9	79.0 ± 24.9	395	< 0.001

Continuous variables are presented as mean ± SD, or median with interquartile ranges. Categorical data are presented as numbers (n) of patients

*Abbreviations*: *eGFR* estimated glomerular filtration rate, *SBP* Systolic blood pressure, *DBP* Diastolic blood pressure, *BMI* Body mass index, *LVH* left ventricular hypertrophy, *ACR* albumin:creatinine ratio, *ALB* Albumin, *Hs-CRP* high-sensitivity C-reactive protein, *TC* Serum cholesterol

**Fig. 1:**
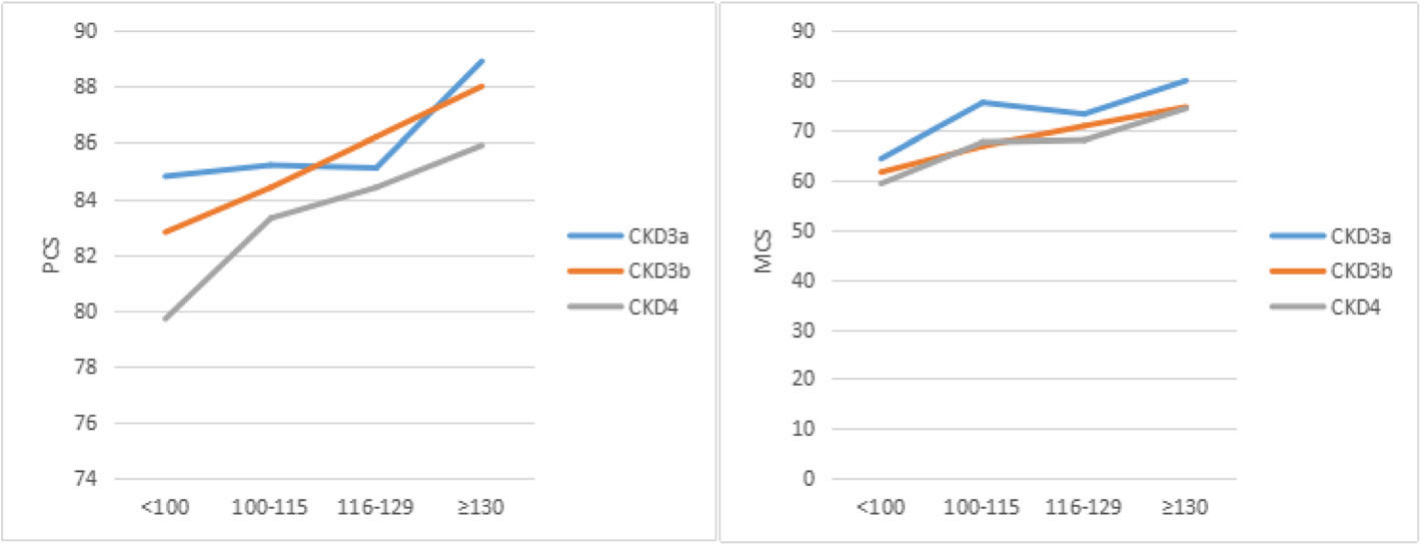
Mean Physical Composite summary (PCS) and Mental Composite Summay (MCS) scores related to declining levels of hemoglobin in CKD

HRQoL Scores by Hb level and CKD stage are presented in Table 2, with the patients divided into three groups according to their renal function; CDK 3a, with GFR range 45–59 ml/min/1.73 m^2^,CKD 3b,with GFR range 30–44 ml/min/1.73 m^2^ and CKD 4, with GFR range 15–29 ml/min/1.73 m^2^. HRQoL scores of CKD deteriorated with the decrease of hemoglobin level. But the change was not significantin hemoglobin level 100–115 g/L and 116–129 g/L,especially PCS and MCS (Fig. 1).

**Table 2:** KDQOL Scores by Hb level and CKD stage

	CKD3a				CKD3b				CKD4			
	< 100	100–115	116–129	≥130	< 100	100–115	116–129	≥130	< 100	100–115	116–129	≥130
Symptoms and Problems (S)	75.0 ± 4.6	77.2 ± 4.0	80.7 ± 2.6	84.7 ± 1.4	73.2 ± 4.3	78.3 ± 2.5	81.8 ± 1.7	81.1 ± 1.6	71.7 ± 1.2	76.6 ± 1.8	80.8 ± 1.7	78.8 ± 2.0
Effects of the Kidney Disease (E)	60.0 ± 5.3	64.3 ± 3.3	63.3 ± 2.8	65.2 ± 1.6	56.6 ± 3.7	57.9 ± 2.6	63.3 ± 2.0	63.4 ± 1.7	54.5 ± 1.9	57.4 ± 2.0	60.2 ± 2.1	62.0 ± 2.3
Burden of the Kidney Disease (B)	76.7 ± 3.3	76.6 ± 2.9	75.1 ± 2.0	77.3 ± 1.1	69.4 ± 3.2	71.9 ± 2.0	72.0 ± 1.5	74.1 ± 1.2	65.6 ± 1.6	68.1 ± 1.5	70.4 ± 1.5	73.9 ± **1.6**
SF-12 Physical Functioning (PCS)	84.8 ± 5.9	85.2 ± 2.1	85.1 ± 1.1	88.9 ± 0.7	82.8 ± 2.3	84.4 ± 1.3	86.2 ± 1.0	88.0 ± 0.8	79.7 ± 1.2	83.3 ± 0.9	84.4 ± 1.0	85.9 ± 1.2
SF-12 Mental Functioning (MCS)	64.3 ± 6.3	75.5 ± 4.1	73.2 ± 2.6	79.9 ± 1.6	61.6 ± 4.4	66.8 ± 2.6	70.8 ± 2.1	74.6 ± 1.8	59.3 ± 2.2	67.6 ± 2.0	68.0 ± 1.8	74.3 ± 2.1

In unadjusted analyses (model 1), HRQoL scores in patients with CKD decreased sharply as hemoglobin declined to 100 g/L,After adjusting for sociodemographics (model 2) and additionally for cardiovascular risk factors (model 3), the degrees of decrease became somewhat blunted. In the final model (model 4), further adjusted for comorbid conditions, these adjusted means also decreased progressively as hemoglobin level declined (Table 3).

**Table 3:** Adjusted Mean Differences of KDQOL Scores Among Different Levels of Hemoglobin

Model	< 100(n = 300)	100–115(n = 525)	116–129(n = 716)	≥130(n = 1380)
Symptoms and Problems (S)				
Model 1	−0.104(− 0.105,-0.102)	− 0.048(− 0.049,-0.046)	− 0.032(− 0.033,-0.031)	ref
Model 2	− 0.083(− 0.085,-0.082)	− 0.031(− 0.032,-0.030)	− 0.018(− 0.019,-0.017)	ref
Model 3	− 0.074(− 0.078,-0.071)	−0.009(− 0.013,-0.006)	− 0.012(− 0.016,-0.008)	ref
Model 4	−0.047(− 0.049,-0.045)	− 0.014(− 0.015,-0.012)	−0.013(− 0.014,-0.012)	ref
Effects of the Kidney Disease (E)				
Model 1	−0.094(− 0.096,-0.092)	−0.052(− 0.054,-0.051)	−0.027(− 0.028,-0.026)	ref
Model 2	− 0.088(− 0.089,-0.086)	−0.049(− 0.050,-0.047)	− 0.026(− 0.027,-0.025)	ref
Model 3	−0.095(− 0.098,-0.091)	−0.016(− 0.020,-0.012)	−0.026(− 0.030,-0.022)	ref
Model 4	−0.047(− 0.049,-0.044)	−0.020(− 0.021,-0.018)	−0.016(− 0.018,-0.015)	ref
Burden of the Kidney Disease (B)				
Model 1	−0.348(− 0.351,-0.345)	−0.162(− 0.164,-0.160)	−0.058(− 0.060,0.056)	ref
Model 2	−0.304(− 0.307,-0.300)	− 0105(− 0.132,-0.128)	−0.048(− 0.050,-0.046)	ref
Model 3	−0.287(− 0.294,-0.281)	−0.117(− 0.123,-0.110)	−0.075(− 0.081,-0.070)	ref
Model 4	−0.207(− 0.212,-0.203)	−0.061(− 0.064,-0.058)	−0.032(− 0.035,-0.030)	ref
SF-12 Physical Functioning (PCS)				
Model 1	−0.266(− 0.269,-0.264)	−0.196(− 0.198,-0.194)	−0.115(− 0.116,-0.114)	ref
Model 2	−0.179(− 0.181,-0.176)	−0.131(− 0.133,-0.130)	−0.057(− 0.059,-0.056)	ref
Model 3	−0.205(− 0.201,-0.199)	−0.108(− 0.114,-0.103)	−0.082(− 0.088,-0.077)	ref
Model 4	−0.112(− 0.115,-0.109)	−0.047(− 0.049,-0.045)	−0.064(− 0.066,-0 .062)	ref
SF-12 Mental Functioning (MCS)				
Model 1	−0.378(− 0.381,-0.376)	−0.201(− 0.203,-0.199)	−0.143(− 0.145,-0.142)	ref
Model 2	−0.317(− 0.320,-0.315)	−0.165(− 0.166,-0.163)	−0.113(− 0.115,-0.111)	ref
Model 3	−0.334(− 0.341,-0.328)	−0.172(− 0.178,-0.166)	−0.048(− 0.053,-0.042)	ref
Model 4	−0.295(− 0.299,-0.292)	−0.098(− 0.101,-0.096)	−0.101(− 0.103,-0.099)	ref

Note: Values are given as adjusted mean difference (95% confidence interval) of the KDQOL scores

Model 1 was unadjusted

Model 2 was adjusted for age, sex, education, and income

Model 3 was adjusted for model 2 + smoking, BMI, diabetes, and hypertension

Model 4 was adjusted for model 3 + LVH, eGFR

*Abbreviations*: *BMI* Body mass index, *eGFR* estimated glomerular filtration rate, *LVH* left ventricular hypertrophy, *eGFR* estimated glomerular filtration rate

## Discussion

Understanding the epidemiology of hemoglobin level in persons with CKD is important when considering the development and implementation of effective prevention and treatment strategies. This study is the first extensive cohort in China. Our findings demonstrate that among persons with CKD, the prevalence of lower hemoglobin increases linearly as eGFR declines and that this association is consistent across age, sex, race, and other clinically important patient groups.

Several studies have documented a high prevalence of anemia in patients with chronic kidney disease. However, most of these studies were regional. We used a standard design for a population survey and a strict quality control procedure to ensure the representativeness of our study. In this cohort of patients with CKD, the prevalence of hemoglobin<100 g/L was 10.3%, and also detected that the prevalence increased with decreasing level of eGFR (from 2.7 to 23.4% when eGFR decreased from CKD stage 2 to CKD stage 4). Similar patterns were observed in other study. In the US,McClellan et al. reported that prevalence of 47.7 and 8.9% for hemoglobin≤120 g/L and hemoglobin≤100 g/L, respectively [15] .The prevalence was comparable to that reported of urban Chinese patients (52.8% for hemoglobin≤130 g/L and 10.3% for hemoglobin≤100 g/L).

In our study, 34.0% of patients with hemoglobin< 100 g/L were under treatment with either ESA or iron, 24.0% of patients using ESA and 24.7% of patients using iron (including both intravenous and oral iron). But the majority of patients were not treated. With the decrease of eGFR, the treatment rate increased gradually. According to the current clinical guideline, the recommended hemoglobin target is 100-110 g/L [16]^.^ There were 11.2% of patients with hemoglobin of 116-129 g/L and 1.7% with hemoglobin≥130 g/L using anti-anemia treatment. It is unknown whether the treatment is due to aims of maintaining an appropriate hemoglobin level or misuse of treatment. We observed largely similar proportion for the treatment of ESA or iron through all the hemoglobin levels, as well as after being stratified by eGFR levels. However, we found that prevalence of iron use was higher than ESA use. The treatment pattern may be due to the primary or initial intervention in these patients.

In the past years, clinical trials have shown that higher hemoglobin treatment targets and/or use of high ESA doses may increase cardiovascular risk for CKD patients [17–20]. These findings may help to reduce the prescribed ESA dose [21] and target hemoglobin levels [16, 22]. However, complex pathogenesis processes other than anemia itself may contribute to the adverse outcomes for patients with CKD [23]. For example, Hung and colleagues found that anemia together with fluid retention instead of only anemia was associated with adverse outcomes of CKD [2]. Nonetheless, higher hemoglobin targets for some patients (that is, individualization of treatment) continues to be discussed and practiced.

Left ventricular hypertrophy (LVH) is considered an important risk factor for adverse cardiovascular (CV) outcomes in patients with chronic kidney disease (CKD), Hemoglobin (Hb) levels have been found to predict the degree of LVH in long-term dialysis patients. Cardiovascular disease (CVD) is the leading cause of morbidity and mortality in patients with chronic kidney disease (CKD). This increased risk begins during the earlier stages of CKD before the onset of kidney failure. Our results show that among CKD patients, the prevalence of LVH among all patients was 7.8%, The next analysis demonstrated variety in LVH with changes in hemoglobin level among patients with CKD, which is consistent with crosssectional studies of patients with equivalent levels of renal dysfunction. Our results show that hemoglobin less than 100 g / L increases the incidence of left ventricular hypertrophy in CKD patients, and indicating an increased cardiovascular risk in this group.

Although monitoring biomarkers is central to the successful treatment of patients with CKD, it is necessary but insufficient to understand fully patients’ overall burden of illness. HRQoL is an important outcome measure in patients with chronic kidney disease. It also has been shown repeatedly to predict mortality in various patient populations. We conducted this systematic review first to establish which domains of HRQOL are most affected by hemoglobin in CKD and then to pool data to measure the magnitude of relationships between traditional biomarkers, including those supported by Kidney Disease Outcomes Quality Initiative (KDOQI) guidelines and tracked by Medicare, and patient-reported HRQOL.

Our study has several key findings. First, we assessed the association between HRQoL and different hemoglobin level in CKD. Present results indicate that HRQoL dimensions were significantly impaired across CKD stages. As expected,the lowest HRQoL scores were seen in the patients with the most declined hemoglobin level. Specifically, the HRQOL decrement in CKD is most pronounced in physical function and vitality. In contrast, the impact of hemoglobin decreased in CKD is pronounced in mental health. This shows that the mental burden of CKD patients is heavy when hemoglobin is decreased, and the quality of life can be significantly improved by increasing hemoglobin.

Second, we found that although the quality of life increased with hemoglobin levels, the KDQOL scores between 100 and 115 and 116–119 showed a downward trend. Compared to hemoglobin levels in the 100–115 group, the quality of life score in the CKD3–4 group of hemoglobin 115–129 did not improve significantly, especially in PCS and MCS. The findings are likely to be related to small differences between groups in HRQoL scores, but they also suggest that keeping hemoglobin levels above 100 g/L may be physical and mental function in CKD patients.

Last,in the multivariable model with adjustment for traditional cardiovascular risk factors and kidney function, lower hemoglobin levels were consistently associated with lower KDQOL scores. Although this finding suggests that improvement of hemoglobin levels can be associated with a better quality of life, a recently published meta-analysis of 17 randomized trials involving 10,049 patients (7616 pre-dialysis CKD patients, 2387 hemodialysis recipients, and 46 peritoneal dialysis recipients) did not support a significant improvement in quality of life with ESA therapy targeting higher hemoglobin levels [24]. However, it is noteworthy that the individual study findings included in the meta-analysis were highly heterogeneous and few of them were categorized as high quality. Hence, more high-quality clinical trials are warranted to determine the efficacy of anemia treatment on the patient-relevant outcomes such as HRQoL.

Although our study bears a large sample size, simultaneous assessment between hemoglobin and KDQOL and investigation of major types of treatment for anemia, there are some limitations still. First, our study was based on the baseline survey of the cohort study, thus the cross-sectional analysis cannot elucidate causal relationships. Second, KDQOL was assessed only once and cannot reflect the dynamic status of patients. Third, the study participants were recruited from outpatient clinics in large clinical centers around China and the majority of etiology was glomerulonephritis. Thus, the generalizability to other populations will be limited.. Also, mild CKD (Stages 1, 2) made up 1/3 of the study cohort, so that anemia prevalence may be higher if these are excluded then the patients with CKD Stage 5 included. The lower QoL scores showed up, the more treatments would be likely given.

## Conclusions

In summary, our study reported the prevalence of different levels of hemoglobin and treatment against anemia in a cohort of Chinese patients with CKD. Increased prevalence of anemia and treatment was associated with decreased eGFR. KDQOL scores in all five domains were reduced through the decreasing level of hemoglobin and the trend was independent of traditional cardiovascular risk factors and kidney function. More observational studies are needed to confirm this finding, and randomized clinical trials are needed to explore the target of hemoglobin in the treatment of CKD-related anemia to improve patient-centered results.

## Data Availability

The datasets used and/or analysed during the current reasch are available from the corresponding author on reasonable request. The datasets generated during and/or analysed during the current study are available from the corresponding author on reasonable request.

## Abbreviations

CKD: Chronic kidney disease
HRQoL: Health-related quality of life
KDQOL-TM: Kidney Disease Quality of Life Short Form
C-STRIDE: Chinese Cohort Study of Chronic Kidney Disease
MDRD: Modification of Diet in Renal Disease
HGB: Hemoglobin- Serum hemoglobin
PCS: SF-12 Physical Functioning
MCS: SF-12 Mental Functioning
UACR: urinary albumin-creatinine ratio
LVH: left ventricular hypertrophy
IVST: interventricular septum
PWT: left ventricular posterior wall
